# Renal effects of angiotensin II in the newborn period: role of type 1 and type 2 receptors

**DOI:** 10.1186/s12899-016-0022-3

**Published:** 2016-04-18

**Authors:** Angela E. Vinturache, Francine G. Smith

**Affiliations:** Department of Physiology & Pharmacology; Alberta Children’s Hospital Research Institute for Child and Maternal Health, Cumming School of Medicine, University of Calgary, 3330 Hospital Drive, NW, Calgary, AB T2N 4N1 Canada

**Keywords:** Newborn, Kidney, Angiotensin receptor type 1, Angiotensin receptor type 2, Angiotensin II, Electrolytes, Development

## Abstract

**Background:**

Evidence suggests a critical role for the renin-angiotensin system in regulating renal function during postnatal development. However, the physiological relevance of a highly elevated renin-angiotensin system early in life is not well understood, nor which angiotensin receptors might be involved. This study was designed to investigate the roles of angiotensin receptors type 1 (AT1R) and type 2 (AT2R) in regulating glomerular and tubular function during postnatal development.

**Methods:**

The renal effects of the selective antagonist to AT1R, ZD 7155 and to AT2R, PD 1233319 were evaluated in two groups of conscious chronically instrumented lambs aged ~ one week (*N* = 8) and ~ six weeks (*N* = 10). Two experiments were carried out in each animal and consisted of the assessment of renal variables including glomerular and tubular function, for 30 min before (Control) and 60 min after infusion of ZD 7155 and PD 123319, respectively. Statistical significance was determined using parametric testing (Student *t*-test, analysis of variance ANOVA) as appropriate.

**Results:**

ZD 7155 infusion was associated with a significant decrease in glomerular filtration rate and filtration fraction at one but not six weeks; urinary flow rate decreased significantly in older animals, whereas sodium excretion and free water clearance were not altered. There was an age-dependent effect on potassium handling along the nephron, potassium excretion decreasing after ZD 7155 infusion in younger but not in older lambs. PD 123319 had no significant effects on glomerular filtration rate and tubular function in either age group.

**Conclusions:**

These results provide evidence to support an important role for AT1Rs in mediating the renal effects of angiotensin II during postnatal maturation in conscious developing animals. In contrast to a role for AT2Rs later in life, there appears to be no role for AT2Rs in influencing the renal effects of Angiotensin II in the postnatal period.

**Electronic supplementary material:**

The online version of this article (doi:10.1186/s12899-016-0022-3) contains supplementary material, which is available to authorized users.

## Background

The marked adaptive changes in renal function, including renal perfusion pressure, glomerular filtration [4, 35] and tubular function [5, 7, 10, 22, 24, 25, 44], at the time of birth may be mediated through complex temporal and spatial alterations in the level of expression and functionality of various factors, among which the renin-angiotensin system (RAS) may play a major role. All RAS components are highly expressed in numerous tissues and organs including the developing kidney of the fetus and the newborn and decrease gradually over the first months in an age-dependent manner (reviewed by Chen et al. (2004) [15]. Studies in mice, rats, sheep, swine and humans have shown that the expression and distribution of the angiotensin receptors (ATRs), type 1 (AT1R) and type 2 (AT2R), is developmentally regulated: A shift in the expression of ATRs subtypes occurs within the kidney, including the renal vasculature, from the AT2Rs, which predominate during fetal life, to AT1Rs whose expression is low in early gestation and increases with age to be the predominant receptor subtype expressed in the adult [1, 2, 6, 20, 49, 50, 63]. Previous studies from us in lambs [56] and from others in developing swine [6], human and simian kidney [63] support the dogma of altered intra-renal expression of ATRs genes with maturation.

Although strong evidence suggests the integrity of the AT1R signalling pathway is a pre-requisite for normal renal development [15, 28], the physiological effects mediated by activation of AT1Rs and/or AT2Rs during ontogeny are not well characterized [58].

Previous studies have shown that during the first six weeks of postnatal life, the pressor and vasoconstrictor effects of Angiotensin II (Ang II) are mediated through exclusive activation of AT1Rs in conscious chronically instrumented lambs [59]. Neonatal treatment with ACE inhibitors or AT1Rs antagonists produces irreversible renal anomalies and alters glomerular and tubular function in the newborn of several species, including the human [21, 26, 27, 37, 40, 43]. To date, however, the physiological relevance of renal AT2Rs during postnatal maturation is not known.

Therefore, the present study aims to address this gap in knowledge and investigate the roles of AT2Rs in regulating renal function in the developing kidney. Based on the anatomical evidence, we hypothesised that, under physiologic conditions, AT2Rs are more important in regulating renal function in the newborn, whereas AT1Rs predominantly regulate glomerular and tubular function later in life. To this end, we measured the responses to administration of selective antagonists to AT1Rs, ZD 7155, and to AT2Rs, PD123319, on various parameters of glomerular and tubular function in two groups of conscious chronically instrumented lambs aged ~ one week, newborns, and later in life, ~six weeks after birth.

## Methods

### Animals, maintenance and diet

Experiments were carried out in two age groups of conscious, chronically instrumented lambs: newborn lambs, aged ~ one week (8 ± 1 days; 7.4 ± 0.2 kg body weight; *N* = 8), and older animals, aged ~ six weeks (41 ± 1 days; 14.8 ± 0.1 kg body weight; *N* = 10). The choice of age reflects age-dependent major physiological changes that we have previously documented regarding baseline systemic and renal haemodynamics [21, 47], circulating levels of Ang II [39, 42], and the responsiveness of the systemic vasculature to vasoactive agents [45, 51].

Lambs were obtained from a local source (Woolfitt Acres, Olds, Alberta) and housed with their mothers in individual pens in the *vivarium* of the Health Science Center except during surgery, training and experiments. Lambs were allowed to suckle *ad libitum*. The lactating ewes were provided with equilibrated diet of proteins, fat, crude fibers, minerals and vitamins and tap water *at libitum*.

All surgical and experimental procedures from this study were carried out with the approval of the Animal Care Committee at the University of Calgary, in accordance with the “Guide to the Care and Use of Experimental Animals” provided by the Canadian Council on Animal Care.

### Surgical procedures

Surgery was performed on lambs using aseptic techniques as previously described [46, 59]. Following induction of isoflurane anaesthesia, catheters were inserted into left and right femoral arteries and veins (Tygon Microbore Tubing CO., USA) and advanced to the level of abdominal aorta and inferior vena cava, respectively for later infusion of drugs and fluids, arterial sampling and pressure measurements during experiments. Through a midline abdominal incision, the bladder was exposed and a catheter (adapted from a feeding tube, Medi-Craft Ltd., Malton, Ontario, Canada) was inserted directly across the bladder wall to be used for measurements of urinary flow rate and urine sample collection during experiments. A left flank incision exposed the left kidney and a pre-calibrated ultrasonic flow transducer (size 3-6S, Transonic Systems Inc., NY, USA) was placed around the renal artery for continuous measurements of renal blood flow during experiments. The incisions were closed and all catheters tunneled subcutaneously to exit the animal on the left and right flanks and secured inside pockets in a lamb body jacket (Lomir Inc., Montreal, Canada).

After surgery, animals were allowed to recover from the effects of anaesthesia and surgery inside a Shor-Line intensive care unit for small animals (Schroer Manufacturing Company, Kansas, USA) with adjustable temperature and oxygen supply. Lambs were returned to the ewe and closely monitored until suckling resumed. Antibiotic, Excenel® (Ceftriofur) 2.2 mg/kg, (Pfizer, Kirkland, QC, Canada), was administered intramuscularly prior to surgery and at 24 h intervals for the following 48 h. For at least four days of recovery after surgery, the lambs were trained for approximately one hour each day to rest comfortably in a supportive sling in the laboratory environment.

### Experimental details

On the day of an experiment, each animal was transported to the laboratory and placed in the supportive sling. At least 60 min were allowed for the animal to become accustomed to its surroundings, during which a pre-warmed intravenous (I.V.) infusion of 5 % dextrose in 0.9 % sodium chloride administered at a rate of 4.17 mL · kg^−1^ · h^−1^ through the right femoral venous catheter was initiated and continued for the duration of the study in order to assist fluid and electrolyte balance. During this time the bladder was allowed to drain. A priming dose of lithium chloride was injected slowly (over ten seconds) 30 min before starting the experiments as a bolus injection of 200 μm∙kg^−1^ for later determination of proximal tubular Na^+^ reabsorption, as previously described [54, 55]. The left femoral venous and arterial catheters were connected to pressure transducers (Model P23XL, Statham, West Warwick RI, USA) for monitoring venous and arterial pressures, respectively. The flow transducer placed around the renal artery was connected to a flow meter (T101, Transonics Systems, Ithaca, NY) for measurement of renal blood flow (RBF). Haemodynamic variables were recorded onto a polygraph (Model 7, Grass Technologies, Astro-Med Inc., West Warwick, RI, USA) and simultaneously digitized at 200 Hz using the data acquisition and analysis software package, PolyVIEW™ (AstroMed Inc., Grass Technologies, Astro-Med Inc., West Warwick, RI, USA).

Two experiments were conducted in random order in each animal at 48 h intervals and consisted of a control period for 30 min (Control) followed by I.V. infusion for 60 min of either the angiotensin receptor antagonist, ZD 7155 (experiment one) or PD 123319 (experiment two) (Tocris Bioscince, Tocris Cookson Inc, Ellisville, MO, USA). Both drugs were administered as an I.V. infusion of 70 μg · kg^−1^ · h^−1^ following an I.V. bolus of 100 μg · kg^−1^ using an infusion pump (Microinfusion pump MI 60-1B, World Precision Instruments, Sarasota, Fl, USA). These doses were carefully selected from previous dose–response experiments [14].

Urine was collected continuously and sampled at 30 min intervals. At the end of each collection period, urinary flow rate (V) was recorded and urine samples stored at −70 °C for later measurement of urinary electrolytes (Na^+^, K^+^, Li^+^, Cl^−^), urinary creatinine concentration and urinary osmolality (UOsm). At the midpoint of each 30 min urinary collection, blood samples (3.5 mL) were removed from the femoral arterial catheter. Whole blood (0.5 mL) was used for immediate measurement of haematocrit (Hct) using a microhaematocrit centrifuge (Clay Adams, Parsippany, NJ, USA). The remainder was placed into a chilled heparinised tube and immediately centrifuged; the supernatant was removed and stored at −70 °C for later measurement of the plasma concentrations of electrolytes (Na^+^, K^+^, Cl^−^), plasma creatinine concentration and plasma osmolality. The volume of blood drawn was replaced with equal volumes of 5 % dextrose in 0.9 % NaCl to minimise the haemodynamic effects of sampling.

At the end of the experiments, lambs were euthanised with a lethal dose of barbiturate. The placement of the catheters was verified and the zero offset of the flow transducer was measured. Both kidneys were removed, examined grossly, and weighed to normalise measurements between the two age groups.

### Analytical procedures

Urinary and plasma electrolytes (Na^+^, K^+^, Li^+^, Cl^−^), were measured on thawed samples by ion chromatography (IC 680, Methrom AG, Herisau, Switzerland). Urinary and plasma osmolalities were measured using a micro-osmometer (2430 Multi-OSMETTE™, Precision Systems Inc., Natic, MA, USA). Creatinine concentrations in urine and plasma were measured using a commercially available creatinine assay kit (QuantiChrom Creatinine assay kit, BioAssays Systems, Hayward, CA). Electrolyte concentrations in urine and plasma were used to calculate excretion rates, clearances, and reabsorptions. Creatinine measurements in urine and plasma were used to estimate glomerular filtration rate. Osmolality measurements in urine and plasma were used to calculate osmolar clearances and tubular water handling.

### Data handling and analyses

Renal plasma flow (RPF) was calculated using the formula: RPF (mL/min) = [1 - (RBF x Hct)]. GFR was estimated as creatinine clearance: GFR = (UCr x V)/PCr. Filtration fraction (FF) was determined as GFR/RPF. Clearances of electrolytes (X) were calculated as follows: CX = (UX x V)/PX, where U and P refers to urinary and plasma concentrations of the electrolyte X and V is urinary flow rate. Fractional reabsorption (FR) of electrolytes was calculated from the ratio of electrolyte clearance to GFR: FRx (%) = [1- (CX/GFR)] x100. Proximal tubular Na^+^ reabsorption was assumed equal to fractional Li^+^ clearance (CLi/GFR). Free water clearance was calculated as the difference between urinary flow rate (V) and osmolar clearance. The ratio of urinary Na^+^ concentration to urinary K^+^ (UNa/UK) as well as the transtubular K^+^ gradient (TTKG) were also calculated (TTKG = (UK/PK)/(UOsm/POsm), where UOsm/POsm is the ratio of UOsm to POsm and UK and PK are concentration of K^+^ in urine and respectively plasma).

Renal haemodynamic variables and parameters of renal function were normalized per gram kidney weight to allow comparisons between the two different age groups since kidney weights were different at one and six weeks (Table 1).

**Table 1:** Baseline demographic, haemodynamics, plasma and renal variables in conscious lambs

	One week	Six weeks
Animal characteristics		
Sample size, n	8	10
Sex	3♀/5♂	7♀/3♂
Age, days	8 (1)	41 (1)*
Kidney weight, g	62.9 (15.1)	80.6 (7.7)*
Baseline haemodynamics		
MAP, mmHg	72 (7)	77 (5)*
RBF, mL · g^−1^ · min^−1^	1.7 (0.6)	4.0 (1.6)*
RVR, mmHg · g^−1^ · min^−1^	44.7 (16.6)	19.1 (7.2)*
Baseline renal variables		
RPF, mL · g^−1^ · min^−1^	1.07 (0.33)	5.60 (2.01)*
GFR, mL · g^−1^ · min^−1^	0.39 (0.16)	0.37 (0.20)
FF, %	39.2 (22.8)	18.24 (11.6)*
V, μL · g^−1^ · min^−1^	4.6 (1.5)	4.9 (3.4)
CH_2_O, μL · g^−1^ · min^−1^	−3.7 (2.8)	−8.3 (5.2)*
POsm, mOsm/kgH_2_O	303 (9)	301 (8)
UOsm, mOsm/kgH_2_O	627 (201)	835 (289)*
COsm, μL · g^−1^ · min^−1^	7.9 (3.4)	10.6 (3.5)*
Plasma concentrations		
PNa, mmol · L^−1^	143.2 (5.5)	144.8 (9.3)
PK, mmol · L^−1^	3.5 (0.3)	3.7 (0.4)
PCl, mmol · L^−1^	65.4 (2.4)	64.7 (3.5)
Urinary excretion rates		
UNaV, μmol · g^−1^ · min^−1^	0.05 (0.06)	0.05 (0.03)
UKV, μmol · g^−1^ · min^−1^	0.51 (0.26)	0.84 (0.23)*
UClV, μmol · g^−1^ · min^−1^	0.13 (0.12)	0.16 (0.11)
UNa/UK	0.07 (0.05)	0.08 (0.05)
TTKG	17.8 (6.9)	16.7 (3.1)
Renal clearances		
CNa, μL · g^−1^ · min^−1^	0.2 (0.1)	1.1 (0.6)*
CCl, μL · g^−1^ · min^−1^	2.0 (1.9)	2.4 (1.4)
CK, mL^−1^ · min^−1^	0.15 (0.1)	0.63 (0.3)*

Values are presented as mean  (SD). *p < 0.05 six weeks compared with one week

*MAP* mean arterial pressure, *RBF* renal blood flow, *RVR* renal vascular resistance, *GFR* glomerular filtration rate, *RPF* renal plasma flow, *FF* filtration fraction, *V* urinary flow rate, *PX* plasma concentration of electrolyte X, *UXV* urinary excretion rate of electrolyte X, *CX* clearance of electrolyte X, *UNa/UK* urine Na^+^ to K^+^ ratio, *TTKG* transtubular K^+^ gradient, *UOsm* urinary osmolality, *COsm* clearance of osmoles, *CH*_*2*_ *O* free water clearance

### Statistical analyses

Data were tested for normal distribution before statistical procedures were applied. Student’s unpaired *t*-test was used to compare baseline measurements between the two groups. Two-way analysis of variance (ANOVA) procedures for repeated measures over time with factors age and treatment followed by Holm Sidak multiple comparisons were appropriate were used to compare the effects of the treatments between the groups. The Bonferroni correction was used to adjust for multiple comparisons. Significance was accepted at the 95 % confidence interval. Data analysis was carried out using IBM SPSS statistical software (IBM SPSS Statistics for Windows, version 20.0, IBM Corp., Armonk, NY). All data were expressed as mean ± SD.

## Results

### Baseline parameters

The demographic characteristics and baseline variables of the two groups of lambs are presented in Table 1. Resting renal haemodynamics variables were significantly different between the groups; RVR was lower, whereas RBF was at higher at six weeks than at one week. There were no significant differences in baseline GFR when normalized per kidney weight between the two age groups. RPF and UOsm were significantly lower whereas FF was higher at one week as compared to six weeks. UKV and UClV were similar in both age groups, while CK and CNa were higher at six weeks.

### Renal effects of the AT1R antagonist, ZD 7155

Infusion of ZD 7155 significantly altered systemic and renal haemodynamics variables in one and six week old lambs. As shown in Additional file 1: Table S1, arterial pressure fell by approximately 10 % within 10 min of infusion in both age groups of lambs, from 73 ± 6 mmHg to 63 ± 6 mmHg in one week old lambs and from 77 ± 5 to 69 ± 5 mmHg in six weeks old lambs (*p* < 0.001). Within 10 min of ZD 7155 infusion, RVR decreased in both age groups, whereas RBF increased from 4.1 ± 1.8 to 5.0 ± 2.1 ml · min^−1^ · g^−1^ (*p* < 0.001) in older lambs and did not change in younger ones.

**Fig. 1:**
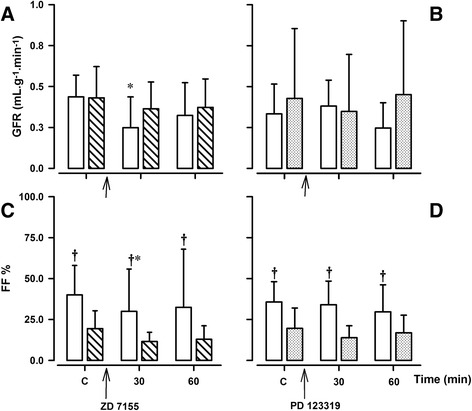
Effects of ATR antagonists, ZD 7155 and PD 123319, on glomerular function in conscious lambs. Effects of ATR antagonists, ZD 7155 and PD 123319 on glomerular filtration rate (GFR; panel **a** and **b**), and filtration fraction (FF; panel **c** and **d**) in conscious lambs aged ~ one week (*open bars*) and ~ six weeks (*closed bars*) measured before (Control, **c**) and for 60 min after intravenous infusion of ZD 7155 (panels **a** and **c**, *striped line bars*) or PD 123319 (panels **b** and **d**, *gray shaded bars*). *p < 0.001 compared to C; †*p* < 0.05 compared to one week

After ZD 7155 infusion, RPF increased in six but not in one week old lambs, from 5.7 ± 2.2 to 6.5 ± 2.5 ml · min^−1^ · g^−1^ (*p* = 0.018). There was an age-dependent effect of ZD 7155 on GFR (F = 3.911, *p* = 0.031), which decreased in one week old lambs from 0.43 ± 0.13 ml · min^−1^ · g^−1^ to 0.24 ± 0.18 ml · min^−1^ · g^−1^ (*p* = 0.010). ZD 7155 did not significantly alter GFR in older lambs, although GFR tended to decline in this group as well, albeit to a lesser extent (from 0.43 ± 0.19 ml · min^−1^ · g^−1^ to 0.37 ± 0.16 ml · min^−1^ · g^−1^) (Fig. 1). Following the changes in RPF and GFR, ZD 7155 elicited age-dependent effects on FF (F = 5.889, *p* = 0.009). FF decreased from 48 ± 25 to 30 ± 26 % (*p* = 0.002) at one week and from 20 ± 10 to 12 ± 8 % at six weeks (*p* = 0.145) (Fig. 1).

**Fig. 2:**
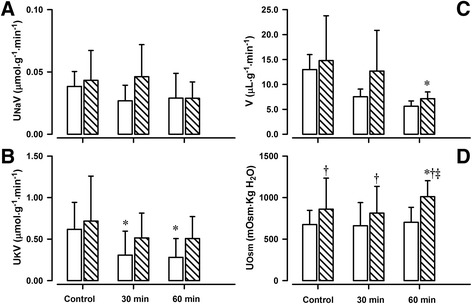
Effects of AT1R antagonist, ZD 7155 on tubular function in conscious lambs. Effects of AT1R antagonist, ZD 7155, on urinary Na^+^ excretion rate (UNaV, panel **a**), urinary K^+^ excretion rate (UKV, panel **c**), urinary flow rate (V, panel **b**), and urine osmolality (UOsm, panel **d**) in conscious lambs aged ~ one week (*open bars*) and ~ six week (*closed, striped line bars*) measured before (Control, **c**) and for 60 min after intravenous infusion of ZD 7155. *p < 0.001 compared to **c**; †*p* < 0.05 compared to one week

Whereas UNaV was not influenced by the ZD 7155 treatment in either age group (Fig. 2), effects on K^+^ excretion were age-dependent (F = 8.391, *p* = 0.001): ZD 7155 significantly decreased UKV in younger animals, from 0.62 ± 0.32 to 0.28 ± 0.22 μmol · min^−1^ · g^−1^ but not in older ones (Fig. 2). This was accompanied by a decrease in TTKG by almost 30 % in younger lambs (*p* < 0.001) (Table 2). There was a trend towards an increase in the UNa/UK ratio after ZD 7155 infusion at one week but not six weeks (*p* = 0.08). There were no significant effects of the AT1R antagonist on Na^+^ and K^+^ reabsorption rates or CK in both groups of lambs (Table 2). CNa was altered by the infusion of ZD 7155 in an age-dependent manner: CNa increased in six but not one week old lambs (F = 28.577, *p* < 0.001).

**Table 2:** Tubular effects of the AT1R antagonist ZD 7155 in conscious lambs

Variable	One week			Six weeks		
	Control	30 min	60 min	Control	30 min	60 min
CNa (μL · g^−1^ · min^−1^)	0.24 ± 0.05	0.18 ± 0.08	0.21 ± 0.16	0.90 ± 0.52**	0.94 ± 0.42**	0.62 ± 0.29**,*
FRNa %	99.93 ± 0.03	99.90 ± 0.08	99.73 ± 0.60	99.90 ± 0.12	99.84 ± 0.23	99.89 ± 0.12
PRNa %	78.3 ± 8.8	81.8 ± 9.1	84.1 ± 10.8	70.4 ± 19.1**	69.4 ± 21.1**	70.1 ± 15.6**
DRNa %	21.7 ± 8.8	18.1 ± 9.1	15.6 ± 10.6	29.5 ± 19.1**	30.6 ± 21.6**	29.8 ± 15.6**
CK (mL · g^−1^ · min^−1^)	0.18 ± 0.10	0.09 ± 0.08	0.09 ± 0.07	0.64 ± 0.34**	0.45 ± 0.16**	0.39 ± 0.16**
FRK %	59.2 ± 17.1	66.0 ± 16.3	64.3 ± 23.7	56.7 ± 18.9	53.5 ± 14.3	57.0 ± 26.0
UNa/UK	0.08 ± 0.03	0.15 ± 0.13	0.12 ± 0.10	0.07 ± 0.04	0.10 ± 0.09	0.10 ± 0.10
TTKG	21 ± 7	17 ± 4*	16 ± 2*	17 ± 3	16 ± 3	16 ± 2**
CCl (μL · g^−1^ · min^−1^)	2.0 ± 1.0	1.2 ± 0.9	0.9 ± 0.5*	2.2 ± 1.0	2.5 ± 1.7	2.6 ± 1.5
FRCl %	99.51 ± 0.24	99.46 ± 0.33	99.31 ± 0.92*	99.29 ± 0.39	99.62 ± 1.82	99.15 ± 0.49**
COsm (μL · g^−1^ · min^−1^)	8.9 ± 1.3	4.6 ± 3.3*	4.2 ± 1.4*	10.1 ± 3.1	8.5 ± 3.3**	8.2 ± 1.9**
UOsm/POsm	1.9 ± 0.5	2.2 ± 0.8	2.2 ± 0.6	2.8 ± 1.2	2.7 ± 1.1	3.1 ± 1.2
CH_2_O (μL · g^−1^ · min^−1^)	−5.1 ± 1.4	−3.1 ± 2.9	−2.3 ± 1.2	−6.9 ± 2.8	−5.7 ± 2.8	−5.6 ± 2.4**

Data are mean ± SD. *p < 0.05 compared to C; **p < 0.05 compared to one week old lambs

*CX* clearance of electrolyte X, *FRX* fractional reabsorption of electrolyte X, *PRX* proximal reabsorption of electrolyte X, *DRX* distal reabsorption of electrolyte X, *UNa/UK* urine Na^+^ to K^+^ ratio, *TTKG* transtubular K^+^ gradient, *UOsm* urinary osmolality, *COsm* clearance of osmoles, *CH*_*2*_ *O* free water clearance

V fell by ~ 50 % within 60 min of ZD 7155 infusion in older lambs (*p* = 0.006). Similar, there was a trend for V to decrease in younger animals; however, this did not reach statistical significance (*p* = 0.058) (Fig. 2).

There were also age-dependent effects of ZD 7155 treatment on UOsm (F = 27.721, *p* < 0.001) and COsm (F = 7.267, *p* = 0.017). UOsm increased in six but not one week old lambs after 60 min of AT1R antagonist infusion (*p* = 0.004) (Fig. 2), whereas COsm decreased in one week old lambs (*p* < 0.001) (Table 2). There were no effects of ZD 7155 on CH2O or the UOsm/POsm ratio in either age group.

### Renal effects of PD 123319

**Fig. 3:**
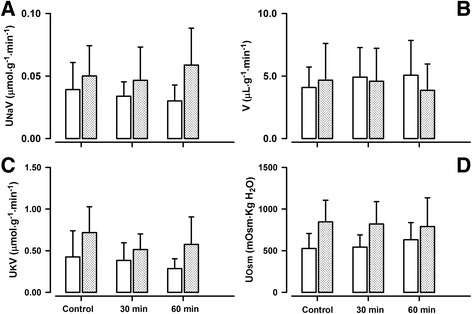
Effects of AT2R antagonist PD 123319 on tubular function in conscious lambs. Effects of AT2R antagonist, PD 123319 on urinary Na^+^ excretion rate (UNaV, panel **a**), urinary K^+^ excretion rate (UKV, panel **c**), urinary flow rate (V, panel **b**), and urine osmolality (UOsm, panel **d**) in conscious lambs aged ~ one week (*open bars*) and ~ six week (*closed, gray shaded bars*) measured before (Control, **c**) and for 60 min after intravenous infusion of ZD 7155. *p < 0.001 compared to **c**; †*p* < 0.05 compared to one week

The AT2R antagonist, PD 123319 had no significant effects on systemic and renal haemodynamics in either group of animals (Additional file 1: Table S1). There were also no changes in RPF, GFR or FF after infusion of PD 123319 (Fig. 1) in either age group. Also, no changes were observed in urine production (Fig. 3) and electrolyte excretion rates of Na^+^, K^+^, (Fig. 3) and Cl^−^ (Table 3) after PD 123319 at one and six weeks. PD 123319 infusion did not alter reabsorption rates and clearances of electrolytes, urine osmolality nor osmolar clearance (Table 3).

**Table 3:** Tubular effects of the AT2R antagonist PD 123319 in conscious lambs

Variable	One week			Six weeks		
	Control	30 min	60 min	Control	30 min	60 min
CNa (μL · g^−1^ · min^−1^)	0.14 ± 0.08	0.23 ± 0.18	0.36 ± 0.44	1.2 ± 0.6**	1.0 ± 0.5**	1.4 ± 1.2**
FRNa %	99.89 ± 0.13	99.85 ± 0.22	99.75 ± 0.30	99.88 ± 0.09	99.86 ± 0.13	99.86 ± 0.14
PRNa %	78.8 ± 11.2	80.1 ± 8.8	77.9 ± 8.3	67.0 ± 20.3**	67.4 ± 20.2**	72.7 ± 21.0**
DRNa %	21.1 ± 11.1	19.8 ± 8.7	21.9 ± 8.1	35.9 ± 19.0**	35.5 ± 18.8**	29.5 ± 20.8**
CK (mL · g^−1^ · min^−1^)	0.11 ± 0.10	0.12 ± 0.06	0.09 ± 0.03	0.62 ± 0.28**	0.47 ± 0.12**	0.42 ± 0.18**
FRK %	66.7 ± 16.2	66.7 ± 15.9	62.1 ± 16.2	52.8 ± 27.6	47.3 ± 24.3	68.1 ± 16.7
UNa/UK	0.07 ± 0.05	0.09 ± 0.06	0.14 ± 0.14	0.09 ± 0.05	0.11 ± 0.09	0.10 ± 0.09
TTKG	15 ± 5	15 ± 5	13 ± 5	17 ± 3	14 ± 2*	13 ± 4*
CCl (μL · g^−1^ · min^−1^)	2.1 ± 2.1	2.5 ± 2.0	2.1 ± 2.0	2.7 ± 1.8	2.4 ± 1.6	2.8 ± 1.5
FRCl %	99.42 ± 0.39	99.45 ± 0.42	99.30 ± 0.66	99.97 ± 0.02	99.97 ± 0.02	99.97 ± 0.02
COsm (μL · g^−1^ · min^−1^)	7.2 ± 3.9	8.1 ± 4.2	7.4 ± 2.8	10.9 ± 3.7	11.4 ± 3.2	11.1 ± 5.4
UOsm/POsm	1.7 ± 0.5	1.7 ± 0.5	2.0 ± 0.7	2.4 ± 0.7	2.8 ± 0.9	2.1 ± 0.7
CH_2_O (mL · g^−1^ · min^−1^)	−3.2 ± 2.7	−3.2 ± 2.4	−2.6 ± 2.1	−7.5 ± 3.3**	−7.1 ± 1.9**	−5.7 ± 3.7

Data are mean ± SD. *p < 0.05 compared to C; **p < 0.05 six weeks compared to one week

*V* urinary flow rate, *CX* clearance of electrolyte X, *FRX* fractional reabsorption of electrolyte X, *PRX* proximal reabsorption of electrolyte X, *DRX* distal reabsorption of electrolyte X, *UNa/UK* urine Na^+^ to K^+^ ratio, *TTKG* transtubular K^+^ gradient, *UOsm* urinary osmolality, *COsm* clearance of osmoles, *CH*_*2*_ *O* free water clearance

Administration of ATRs antagonists, ZD 7155 and PD 123319, had no significant effects on plasma Na^+^, K^+^ and Cl^−^ concentration, nor did it influence plasma osmolality in either of the age groups (Additional file 2: Table S2).

## Discussion

The present study, exploring the potential roles of Ang II receptors type 1, AT1Rs and type 2, AT2Rs in regulating renal function during postnatal maturation, provides new information on the renal effects of ATRs during the newborn period as follows: i) glomerular ultrafiltration is regulated by Ang II through activation of AT1Rs in an age-dependent manner; ii) production of urine is modulated by activation of AT1Rs predominantly later in life; iii) AT1Rs mediate the effects of Ang II of K^+^ handling along the nephron in an age-dependent manner; iv) AT2Rs alone do not appear to mediate any of the glomerular and tubular effects of Ang II in the newborn period. Therefore, our current findings provide new evidence that, early in life, in conscious animals, Ang II regulates both, glomerular and tubular function through predominant activation of AT1Rs but not AT2Rs.

To our knowledge, no previous studies have explored the roles of ATRs in regulating kidney function in newborns in the conscious, undisturbed state. To date, the evidence is limited to the roles of AT1Rs and comes from studies in anaesthetised newborns animals. For instance, in anaesthetised newborn rabbits, the AT1R antagonist, losartan, elicits dose-dependent systemic and glomerular responses [40]. In neonatal rats, chronic subcutaneous administration of DuP 753 decreased arterial pressure but left GFR unchanged [16]. Intra-renal infusion of the AT1R antagonist A-81988, increased RBF in three weeks old piglets, while the effects on glomerular function were similar in young and adult anaesthetised pigs [52]. This is in contrast with transient effect of the selective AT1R antagonist, ZD 7155 on GFR only in newborns lambs observed in the current experiments in conscious lambs. The differences in responses to AT1R inhibition may be a consequence of selectivity, dose and mode of administration of the drugs (systemic vs. intrarenal), or related to variability in terms of age, renal maturation (level of expression and localization of the receptors within kidney regions in different species), as well as the experimental setting (anaesthetised vs. conscious). Anaesthesia has been shown to activate the RAS and it is conceivable that, in the anaesthetised animals, the RAS is up-regulated to levels much higher than that of conscious newborn animals.

Based on the distinct pattern of ATRs distribution in the developing kidney in several species, including porcine, ovine and primate kidney [6, 29, 30, 41, 49, 56, 57, 61, 63], we hypothesised that immediately after birth activation of AT2Rs may contribute to the adaptation of glomerular function in the immediate newborn period. This was not the case. Our study shows that endogenous Ang II effects on glomerular function are mediated entirely through activation of AT1Rs, likely through a predominant effect on efferent arterioles, with no apparent role for AT2Rs. Nonetheless, the contribution of AT2R in regulating vasomotor activity of the intrarenal vasculature cannot be ruled out at this time and remains to be elucidated.

The intriguing observation that the decline of GFR after ZD 7155 was not sustained in newborn lambs despite the continued inhibition of AT1Rs, also lends support to the concept of redundant mechanisms that are, most likely, already in place at birth. Thus, the newborn kidney is probably able to mobilise additional vasoactive factors to maintain glomerular filtration. For example, AT1R inhibition may have revealed the role of other vasoactive factors, such as prostaglandins, bradykinin, or nitric oxide. Previously, we have reported that the renal haemodynamic effects of nitric oxide are regulated by Ang II through activation of both ATRs [57, 60] in an age-dependent manner, with AT2Rs being a more important regulator of renal haemodynamics in the newborn and AT1Rs more predominant later in life [57]. Also, pre-treatment with the cyclooxygenase inhibitor, indomethacin, augments the pressor response to Ang II and modulates the vasomotor effect of Ang II in response to infusion of the nitric oxide inhibitor, L-NAME, thus removing endogenously produced nitric oxide [23]. Taken together, these findings may suggest that AT2Rs may not need to be recruited to assist in regulating glomerular function in the face of the numerous vasodilators already present.

In adulthood, the effects of Ang II in regulating sodium transport within the kidney, ranging from natriuresis to antinatriuresis are mediated through multiple receptors, including AT1Rs and AT2Rs. Much less is known about Ang II contribution to Na^+^ handling in newborn mammals [29, 32, 53]. For example, in adult animals, in which the RAS is activated by dietary salt restriction, treatment with ACE inhibitors elicits a robust increase in water and electrolyte excretion [12, 36]. Therefore, we anticipated that, in newborn lambs, in which the RAS is also activated [9, 34], there would be a significant increase in water and electrolyte excretion after ATRs inhibition. We also predicted that AT2Rs, which are the predominant type of ATRs expressed within the kidney after birth, may play a predominant role in water and electrolyte handling in the newborn. This was not the case. Effects of Ang II on renal handling of water and electrolytes appeared rather to be mediated predominantly through activation of AT1Rs in an age-dependent manner, whereas AT2Rs do not appear to regulate water and salt handling along the nephron in developing animals.

The decrease in K^+^ excretion in young animals reported in the present study may be due to inhibition of aldosterone secretion through a feedback mechanism at the adrenal gland level, or by inhibition of possible direct regulatory mechanisms of Ang II on K^+^ channels along the collecting duct. The decrease in the flow of tubular fluid in this age group may have contributed to a fall in K^+^ secretion and, therefore, a reduction in K^+^ excretion rate. Absence of significant changes in the UNa/UK ratio may suggest that aldosterone levels may not have been altered after ATRs inhibition. Further studies are needed to explore more in depth the effects of ATRs inhibition on aldosterone activity in newborn.

Our findings are in keeping with observations of the renal effects of the AT1R antagonist losartan in adult, salt-depleted animals, in which the RAS is also activated, similar to newborn lambs in the present experiments [3, 8, 17, 19, 62]. For instance, in sodium-depleted anaesthetised adult rats, AT1R antagonists decreased GFR, urine and electrolytes excretion [16, 33], whereas in sodium-depleted conscious and anaesthetised adult dogs, AT1R antagonists increased GFR and elicited a significant increase in urine volume and urinary sodium excretion rate [8, 18, 31]. However, in this species, AT2R inhibition antagonized the vasoconstrictor effects of exogenous Ang II and induced an increase in urine flow and free water formation, suggesting a functional role for AT2Rs in water handling by the adult kidney [18, 31]. Other studies in adult animals have shown a role for AT2R in mediating vasodilation, natriuresis and diuresis [11, 38], albeit these effects were revealed in anesthetised animals, in conditions of RAS activation and AT1Rs inhibition. As such, an age-dependent functional role for AT2R in renal homeostasis awaits confirmation.

### Strengths and limitations

There are several limitations to our study: i) Due to the technical limitations of surgery and postoperative recovery, the effects of ATRs activation immediately after birth, especially within the first few days after birth could not be evaluated; ii) The sample size in this study was predetermined in order to identify significant changes in arterial pressure [13, 59]. Heterogeneity in study designs and lack of power calculations in most of the previous reports that investigated the effects of ATRs in newborns, offered limited information for sample size calculation in our study; iii) For some of the physiologic variables studied, a trend in changes was observed that did not reach statistical significance. This may be altered if the group sizes were considerably larger although our previous evaluations of renal function in these two age groups have revealed significant differences with similar sample sizes [39, 48]; iv) We were not able to describe the continuum of changes in ATRs functions from one to six weeks after birth, thus we could not determine the postnatal age at which the changes in the activity of ATRs occur. On the other hand, as opposed to the previous studies in fetal animals, which provide only a snapshot of the ATRs function at one gestational age, our study evaluated renal effects of ATRs antagonists at two different stages of postnatal development, thus providing an insight into the physiological changes of renal function that occur with the process of maturation and transition to adulthood. Furthermore, our study was conducted in conscious animals, trained to the laboratory environment, and, therefore, the findings represent physiological conditions.

## Conclusions

In summary, the present observations provide evidence for a more important role for Ang II through AT1Rs in governing renal function during the critical period of postnatal development and adaptation to extra-uterine life. AT1Rs are the predominant effectors of Ang II mediated renal effects throughout postnatal maturation, whereas the physiological functions of AT2R in modulating the kidney have not been elucidated. Additional studies are warranted to explore potential roles of the AT2Rs in modulating AT1R-mediated renal responses during ontogeny.

### Ethics

This study received ethics approval from the Animal Care Committee at the University of Calgary, in accordance with the “Guide to the Care and Use of Experimental Animals” provided by the Canadian Council on Animal Care. The experiments were carried out in conformity with ARRIVE guidelines from the National Centre for the Replacement, Refinement & Reduction of Animals in Research.

### Consent to publish

Not applicable.

## Additional files

Additional file 1: Table S1. Haemodynamic effects of AT1R antagonist, ZD 7155 and AT2R antagonist, PD123319 in conscious lambs. (DOCX 38 kb) Additional file 2: Table S2. Effects of AT1R antagonist, ZD 7155 and AT2R antagonist, PD123319 on plasma variables in conscious lambs. (DOCX 20 kb)

## Abbreviations

AngII: angiotensin II
AT1R: angiotensin receptor type 1
AT2R: angiotensin receptor type 2
CH2O: free water clearance
COsm: clearance of osmoles
CX: clearance of electrolyte X
DRX: distal reabsorption of electrolyte X
FF: filtration fraction
FRX: fractional reabsorption of electrolyte X
GFR: glomerular filtration rate
MAP: mean arterial pressure
Osm: osmolality
PRX: proximal reabsorption of electrolyte X
PX: plasma concentration of electrolyte X
RAS: renin-angiotensin system
RBF: renal blood flow
RPF: renal plasma flow
RVR: renal vascular resistance
TTKG: transtubular K+ gradient
UNa/UK: urine Na^+^ to K^+^ ratio
UOsm: urinary osmolality
UXV: urinary excretion rate of electrolyte X
V: urinary flow rate
