# Supplementary material for: Renal effects of angiotensin II in the newborn period: role of type 1 and type 2 receptors
Source: BMC Physiol. 2016 Apr 18;16:3. doi: 10.1186/s12899-016-0022-3 (PMC4835895; doi:10.1186/s12899-016-0022-3)
Supplement: Additional file 1: Table S1. — Haemodynamic effects of AT1R antagonist, ZD 7155 and AT2R antagonist, PD123319 in conscious lambs. (DOCX 38 kb) [file 12899_2016_22_MOESM1_ESM.docx]

**Additional file 1: Table S1. Haemodynamic effects of AT1R antagonist, ZD 7155 and AT2R antagonist PD123319 in conscious lambs**

| **Variable** | **Age group** | **ZD 7155** | | |  | **PD 123319** | | |
| --- | --- | --- | --- | --- | --- | --- | --- | --- |
|  |  | **Control** | **30min** | **60min** |  | **Control** | **30min** | **60min** |
| **MAP, mmHg** | One week | 73±6 | 64±6***** | 63±6***** |  | 73±7 | 73±6 | 71±4 |
|  | Six weeks | 77±5 | 70±5***** | 69±5***** |  | 77±4 | 76±6 | 75±5 |
| **RBF, mL·g^-1^·min^-1^** | One week | 1.6±0.6 | 2.0±0.7 | 2.0±0.6 |  | 1.6±0.4 | 1.6±0.4 | 1.7±0.5 |
|  | Six weeks | 4.1±1.8 | 5.0±2.1***** | 4.7±1.9***** |  | 4.0±1.4 | 4.2±1.8 | 4.1±1.6 |
| **RVR, mmHg·g^-1^·min^-1^** | One week | 45.8±16.9 | 35.9±19.7***** | 35.9±18.2***** |  | 43.6±14.5 | 43.9±14.9 | 41.6±12.7 |
|  | Six weeks | 18.3±7.8 | 15.7±7.5***** | 16.5±7.9***** |  | 19.9±6.9 | 19.2±6.7 | 18.9±6.6 |

Data are mean ± SD. (*) p<0.05 compared to Control.

MAP, mean arterial pressure; RBF, renal blood flow; RVR, renal vascular resistance.
