# Supplementary material for: Renal effects of angiotensin II in the newborn period: role of type 1 and type 2 receptors
Source: BMC Physiol. 2016 Apr 18;16:3. doi: 10.1186/s12899-016-0022-3 (PMC4835895; doi:10.1186/s12899-016-0022-3)
Supplement: Additional file 2: Table S2. — Effects of AT1R antagonist, ZD 7155 and AT2R antagonist, PD123319 on plasma variables in conscious lambs. (DOCX 20 kb) [file 12899_2016_22_MOESM2_ESM.docx]

**Additional file 2: Table S2. Effects of AT1R antagonist, ZD 7155 and AT2R antagonist PD123319 on plasma variables in conscious lambs**

| **Variable** |  | **ZD 7155** | | |  | **PD 123319** | | |
| --- | --- | --- | --- | --- | --- | --- | --- | --- |
|  |  |  |  |  |  |  |  |  |
|  | **Age group** |  |  |  |  |  |  |  |
|  |  | **control** | **30 min** | **60 min** |  | **control** | **30 min** | **60 min** |
|  |  |  |  |  |  |  |  |  |
| **PNa (mmol·L^-1^)** | One week | 142 ± 9 | 143 ± 6 | 139 ± 9 |  | 140 ± 11 | 141 ± 8 | 140 ± 12 |
|  | Six weeks | 144 ± 8 | 141 ± 9 | 141 ± 9 |  | 141 ± 7 | 140 ± 9 | 138 ± 4 |
| **PK (mmol·L^-1^)** | One week | 3.3 ± 0.2 | 3.4 ± 0.2 | 3.2 ± 0.4 |  | 3.3 ± 0.3 | 3.2 ± 0.2 | 3.3 ± 0.3 |
|  | Six weeks | 3.8 ± 0.3† | 3.7 ± 0.4† | 3.8 ± 0.3† |  | 3.7 ± 0.3† | 3.7 ± 0.3† | 3.7 ± 0.3† |
| **PCl (mmol·L^-1^)** | One week | 65±5 | 66±4 | 66±7 |  | 65 ± 7 | 65 ± 5 | 66 ± 6 |
|  | Six weeks | 64 ± 3 | 62 ± 7 | 62 ± 4 |  | 63 ± 5 | 65 ± 4 | 63 ± 5 |
| **POsm (mOsm·kgH_2_O^-1^)** | One week | 304 ± 11 | 298 ± 8 | 305 ± 10 |  | 302 ± 7 | 305 ± 11 | 305 ±11 |
|  | Six weeks | 303 ± 4 | 297 ± 6 | 299 ± 7 |  | 302 ± 9 | 299 ± 7 | 297 ± 9 |

Data are mean ± SD. *p<0.05 compared to C; **†**p<0.05 six weeks compared to one week

ZD 7155, AT1R antagonist; PD 123319, AT2R antagonist

PX, plasma concentration of electrolyte X; POsm, Plasma osmolality
